# Limited immune perturbations in mice exposed to sustained low-dose ionizing radiation

**DOI:** 10.3389/fimmu.2026.1642012

**Published:** 2026-04-20

**Authors:** Bryan Marr, Holly Laakso, Melinda Blimkie, Andrew Cao, Seung-Hwan Lee, Abrar Ul Haq Khan

**Affiliations:** 1 Department of Biochemistry, Microbiology, and Immunology, Faculty of Medicine, University of Ottawa, Ottawa, ON, Canada; 2 Radiobiology and Health Branch, Canadian Nuclear Laboratories Ltd., Chalk River, ON, Canada; 3 Ottawa Institute of Systems Biology, Faculty of Medicine, University of Ottawa, Ottawa, ON, Canada; 4 Centre for Infection, Immunity, and Inflammation, Faculty of Medicine, University of Ottawa, Ottawa, ON, Canada

**Keywords:** ionizing radiation, low-dose radiation, low-dose rate, radiation immunology, RNA-seq

## Abstract

The effects of high-dose ionizing radiation (HDIR) exposure on the immune system are largely understood with consensus, yet there remains a fragmented understanding of the impact of low-dose ionizing radiation (LDIR) on immune homeostasis, especially in sustained exposure conditions. This study investigates the effects of continuous LDIR exposure on the murine immune system, focusing on transcriptomic responses and cellular perturbations following low-dose-rate whole-body *y*-radiation. Female 18-week-old C57BL/6 mice were continuously exposed to low-dose-rate ^60^Co radiation over a period of 7 days, resulting in cumulative absorbed doses of 10 mGy and 100 mGy. Our findings indicate that the LDIR exposure induced, at most, only minimal transcriptomic perturbations to the immune system in C57BL/6 mice. These results suggest a preservation of immune cell homeostasis under the sustained low-dose-rate exposure conditions studied. It contributes to a broader understanding of radiation biology, emphasizing that the effects of LDIR on the immune system can be limited at low-dose-rates in mice.

## Introduction

Ionizing radiation (IR) is widely used across various sectors; it powers vital medical diagnostics, fuels the engines of energy production, drives industrial tools and processes, and is wielded in weapons of mass destruction. As the utilization of this energy increases, the exposure of people, particularly among the growing occupationally exposed workforce, also increases. Therefore, understanding the interplay between ionizing radiation and cellular machinery is imperative.

While the effects of high-dose ionizing radiation (HDIR) exposure are largely understood with scientific consensus, the biological effects associated with low-dose ionizing radiation (LDIR) exposure continue to be contested. LDIR is defined by the National Academies of Science as exposures resulting in absorbed doses below 0.1Gy (100 mGy) (1). Low-dose-rate radiation is typically defined as being under 0.5-0.6 mGy/hr (1, 2). This level of exposure is significantly lower than exposures encountered in medical diagnostics or acute radiation syndrome (ARS) (3). Instead, it is more relevant to therapeutic procedures, natural background radiation, and occupational exposures, such as in the nuclear power industry or space travel (1).

Despite efforts in both epidemiological and experimental studies, the field lacks a unified comprehension of the effects of LDIR on the immune system. HDIR is known to cause significant immune disruptions, including inflammation, hematopoietic syndrome, and lasting alterations in immune homeostasis (4–11). However, it remains uncertain whether the effects of LDIR exhibit similar but more subtle changes or if they produce completely distinct outcomes. Without a thorough understanding of these effects, the potential role of the immune system in contributing to the reported positive excess relative risk ratios for cancer and other diseases following chronic occupational LDIR exposure remains uncertain (12–17).

In total body irradiation (TBI) studies using animals, exposure to low-doses of external X-ray or gamma radiation (<100 mGy) has been shown to cause modulations in immune cell populations. For instance, LDIR exposure has been associated with increased expression of Trp53 and Nrf2 (18–20). A single exposure to 100 mGy TBI can reduce circulating white blood cell counts and splenocytes in some models (19–22). In other models, changes to cytokines, immunoglobulins, or even T cell receptor diversity have been explored as impacts of LDIR exposure, even in the absence of changes to immune cell numbers (20, 21, 23–25). Other studies can be contrasting, for instance, LDIR in another model was shown to reduce apoptosis in the spleen (26, 27). While the literature exhibits considerable variability, LDIR responses generally appear to be transient and of low magnitude, with their downstream implications for immune homeostasis remaining unclear. Furthermore, there is a growing appreciation for the cell-type specific responses to radiation exposure, but that has not been sufficiently explored in the low-dose range (6, 21, 28–30). Studies suggest that LDIR can cause subtle and variable effects on the immune system. However, there remains little understanding of the molecular mechanisms underlying responses across diverse immune cell populations. Furthermore, most studies utilize high-dose rate single exposures; therefore, there exist minimal studies documenting responses to continuous LDIR exposure conditions. Due to subtle and heterogeneous effects, it has been recommended by the National Academy of Science charged with revitalizing low-dose radiation research, that future studies utilize high-resolution, high-throughput omic technologies and bioinformatic analysis to provide more detailed insights (1).

The objective of this study was to characterize the effects of sustained LDIR on the murine immune system. Specifically, we sought to uncover and describe the mechanisms by which different immune cell populations respond to sustained whole-body radiation exposure. In collaboration with the Canadian Nuclear Laboratories (CNL), female mice were exposed to continuous low-dose-rate gamma rays for one week, reaching cumulative low absorbed doses of 10 mGy or 100 mGy. The total body dose of 100 mGy was chosen because it represents the upper limit of “low dose” radiation as defined in the literature (1). By setting this dose as the highest level of exposure, we aimed to investigate potentially, the most intense response within the low-dose range. We hypothesized that sustained low-dose radiation exposure would induce subtle changes to immune system homeostasis, including both cell-type specific and dose-dependent reactions to LDIR.

## Methods

### Mice irradiation and organ dissociation

Eighteen-week-old female C57BL/6 mice (Jackson Laboratories) were housed and irradiated at the CNL (Chalk River, Ontario, Canada). The entire facility, including the irradiation hall and the conventional animal room where control mice were housed, is Specific Pathogen Free. The mice were fed PicoLab Rodent 20 5K75 chow and provided water in bottles. Housing conditions were maintained at a temperature of 20-26 °C, and humidity of 20-80%, with a 12-hour light/dark cycle. Three mice per cage were housed in Animal Care Systems Optimice cages with BlockParty bedding, and during acclimatization, the cages were individually ventilated on an Optimice rack system. During irradiation in the Gamma Beam Facility, the cages were placed on wooden racks, in static caging, with Biofresh™ Performance Bedding. Enrichment materials included an enviropack, nestlet, wooden block, and hut, with food enrichment of Cheerios provided after ear punching and cage changes.

Whole-body gamma irradiation was administered using a Nordion (Ottawa, Ontario, Canada) Gammabeam 150C irradiator equipped with a Cobalt-60 source. The irradiation was continuous for 7 days in the pathogen-free irradiation hall of the CNL (31). Treatment groups were positioned at varying distances from the irradiator to achieve different dose rate exposures. Mice receiving a cumulative dose of 10 mGy (n=6) received an average dose rate of 0.0614mGy/hr. Mice receiving a cumulative dose of 100 mGy (n=6) received an average dose rate of 0.625mGy/hr. The positioning ensured the beam targeted the mid-level of each cage, centered between the two cages. Dosimetry was performed using an Exradin A8 ion chamber and a Supermax electrometer. Control mice (n=6) were housed separately in a conventional animal room.

Following irradiation, mice were euthanized via exsanguination under Isoflurane gas anesthesia, followed by cervical dislocation. Blood samples were collected via cardiac puncture. Mice were then dissected to collect spleen, bone marrow and thymus. Blood samples were immediately analyzed using the Zoetis Vetscan MS5 hematology analyzer. The remaining organs were transported on ice for around 2.5 hours to the University of Ottawa (Ottawa, Ontario, Canada) for further analysis. All animal procedures were approved by the CNL Animal Care Committee and adhered to Canadian Council on Animal Care standards.

### Cell isolation

Spleens were mechanically disrupted by pressing through a 70µm cell strainer (Bio Basic Canada Inc.) using a syringe plunger. The disrupted tissue was suspended in RPMI medium supplemented with 2% fetal bovine serum (FBS) (Gibco™, Canada) and kept on ice. The suspension was centrifuged at 1200 rpm for 10 minutes at 4 °C. The pellet was resuspended in 1 mL of red blood cell (RBC) lysis buffer (Roche) to lyse erythrocytes. Post-lysis, cells were washed with RPMI containing 2% FBS and filtered through a nylon mesh to remove debris. Thymus samples underwent the same mechanical disruption and subsequent processing steps as spleens, with the omission of red blood cell lysis.

Bone marrow cells were obtained from excised femurs. The femurs were cleaned of excess tissue and placed in cold PBS. They were then dried with a Kimwipe to remove remaining connective tissue. Their ends were removed, and marrow was flushed out with a 27g needle and 1 mL of FBS into a microcentrifuge tube. The marrow was collected by repeated flushing and scraping with the needle to ensure complete extraction, and the femurs were discarded when they appeared pale/white. Bone marrow was kept on ice. Cells were then centrifuged at 1200 rpm for 10 minutes at 4 °C, resuspended in RBC lysis buffer, washed with RPMI containing 2% FBS, and filtered through nylon mesh.

### Ex vivo functional assay

For ex vivo NK cell intra-cellular IFN-*y* measurements, freshly derived splenic leukocytes were stimulated with either a combination of IL-2 (100U/ml, obtained from NCI Preclinical Repository, USA) and IL-12 (10 ng/ml) (eBioscience™), or with plate coated anti-NKp46 (BioLegend™) for 1 hr and then incubated in RP-10 medium containing 5 μg/ml brefeldin A (Invitrogen™) for 4 hrs, followed by intracellular staining. For ex vivo T cell intracellular IFN-*y* measurements, freshly derived spleen leukocytes were stimulated with anti-CD3/28 for 16 hrs and then incubated in RP-10 medium containing 5 μg/ml brefeldin A (Invitrogen™) for a further 4 hrs, followed by intracellular staining.

### Flow cytometry

Cell staining was conducted under cold conditions and protected from light to preserve cell integrity and prevent fluorophore photobleaching. Single-cell suspensions (1x10^6^ cells) were aliquoted into a 96-well V-bottom plate and washed in staining buffer (PBS supplemented with 2% HI-FBS). Cells were incubated at 4 °C for 10 minutes in α-CD16/32 (clone 2.4G2 from Bioexpress) to block non-specific binding. Cells were then stained with a cocktail of fluorophore-conjugated monoclonal antibodies in staining buffer and incubated at 4 °C for 25 minutes. Post-staining, cells were washed with staining buffer and fixed with 2% paraformaldehyde (PFA) for 10 minutes at 4 °C. Cells were then washed, centrifuged and resuspended in the staining buffer for acquisition. For intracellular proteins (Gzmb and CD107a), the Cytofix/Cytoperm kit was employed following surface staining. Briefly, cells were washed and centrifuged, then resuspended in BD Cytofix/Cytoperm buffer and incubated for 10–20 minutes at 4 °C. Cells were washed twice with BD Cytoperm Wash buffer and centrifuged. The cells were then incubated with an intracellular antibody cocktail in BD Cytoperm Wash buffer for 25 minutes at 4 °C. Following incubation, cells were again washed with BD Cytoperm Wash buffer and staining buffer, centrifuged, and resuspended in cold staining buffer for analysis. Flow cytometry was performed using an Attune NxT flow cytometer (Attune, ThermoFisher) and analyzed with Kaluza (Beckman Coulter). Antibodies used included: anti-TCRβ (H57-597), anti-CD8 (53-6.7), anti-CD49b (DX5), anti-CD11b (M1/70), anti-NKG2D (CX5) from eBioscience™; anti-CD19 (1D3), anti-CD4 (RM4-5), anti-F4/80 (T45-2342), anti-NK1.1 (PK136), anti-Ki-67 (B56), anti-CD69 (H1-2F3), anti-Ly6C (AL-21), anti-Gr1 from BD Biosciences™; anti-CD43 (1B11) from BioLegend™; and Live/Dead Fixable Yellow Dead Cell Stain from Invitrogen™.

### Bulk RNA-sequencing and bioinformatics

RNA was extracted from isolated splenocytes using Trizol (Thermofisher). RNA integrity was ensured with a minimum RIN cut-off of 6.5. Poly-A enriched stranded cDNA libraries were generated and subjected to 100 bp paired-end sequencing on a NovaSeq6000 at Genome Quebec (Montreal, Canada). Samples were sequenced to a minimum depth of 23E6 reads. FASTQ files were pseudo-aligned to the mouse transcriptome assembly (mm10) using Kallisto (v.0.46.1). Gene-level quantification was performed with Tximport (v1.32.0). Normalization, differential expression, and principal component analysis were conducted using DESeq2 (v1.44.0). Differentially expressed genes were identified using an adjusted p-value threshold of <0.05. Gene set enrichment analysis (GSEA) was performed using fgsea (v1.30). Heatmaps were generated from variance-stabilized data. Lists of radiation-responsive genes were sourced from the Gene Ontology database, specifically from GO:0009314, GO:0071478, and GO:0010212.

### Plots and statistics

Plots were generated using Kaluza (Beckman Coulter), ggplot2 (v3.5.1), ComplexHeatMap (v2.20.0), Seurat (v5.1.0), cowplot (v1.1.3), and ggVenDiagram (v1.5.2). Hematology and flow cytometry statistical analyses were conducted with the Rstatix (v0.7.2) package. Bulk RNA-sequencing analysis was performed using DESeq2 (v1.44.0).

## Results

### Hematological stability in mice following sustained low-dose radiation exposure

In this study, 18-week-old female C57BL/6 mice were housed for 7 days in front of a ^60^Co gamma beam source until cumulative whole-body absorbed doses of 10 mGy and 100 mGy were reached. An untreated (UT) control was housed separately without exposure to radiation. Immediately following the radiation exposure period, the mice were euthanized, blood samples were acquired, and immune cells were isolated from the spleen, thymus, and bone marrow for further study.

**Figure 1:**
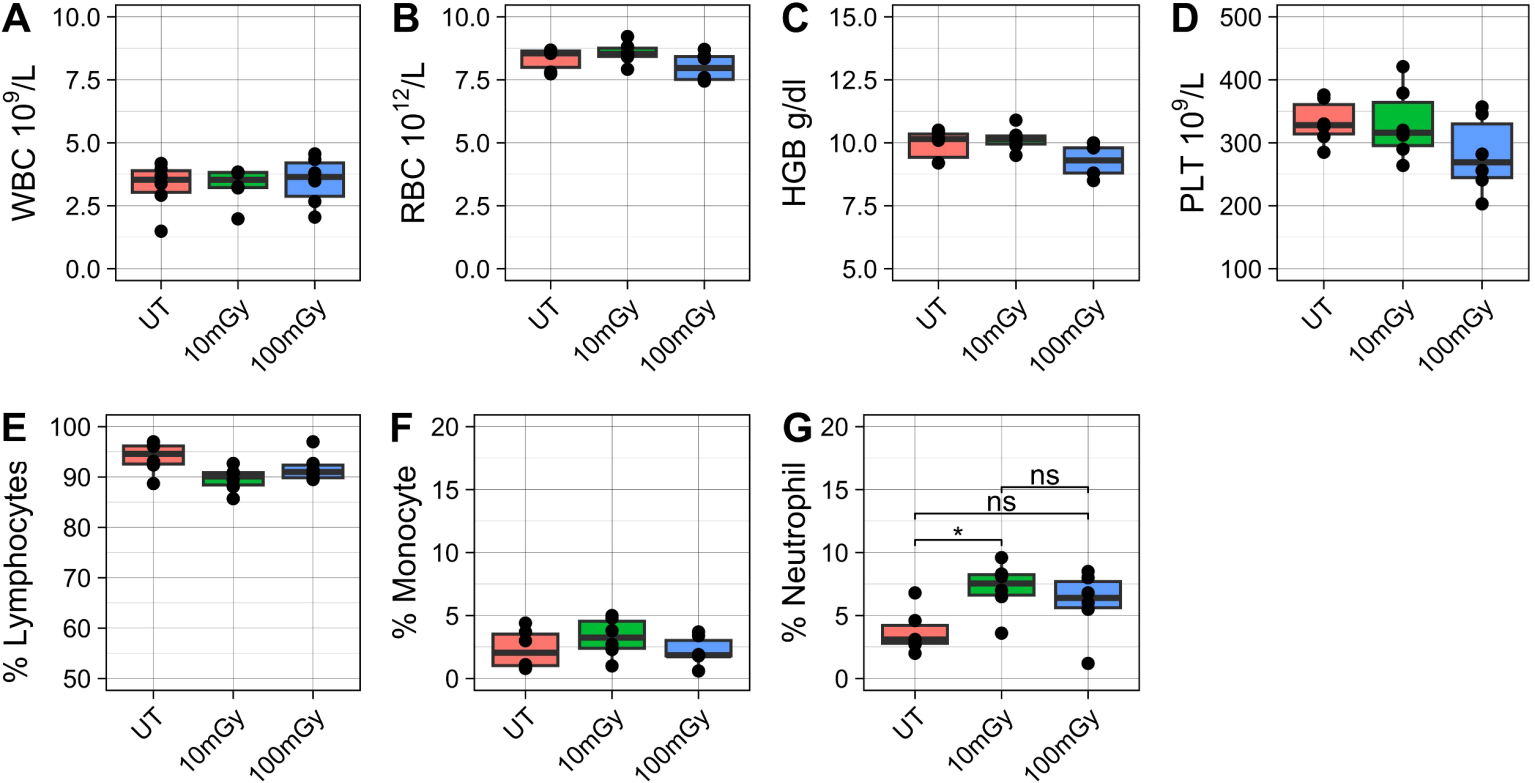
Post-irradiation hematology analysis. Blood samples were collected via cardiac puncture immediately following irradiation and analyzed for hematological parameters. The figure depicts **(A)** white blood cell (WBC) counts, **(B)** red blood cell (RBC) counts, **(C)** hemoglobin (HGB) levels, and **(D)** platelet (PLT) counts, as well as the proportions of **(E)** lymphocytes, **(F)** monocytes, and **(G)** neutrophils among indicated groups of mice. Statistical comparisons were conducted using the Kruskal-Wallis test followed by *post hoc* Dunn's tests.

A complete blood analysis was conducted to assess changes in blood cells, which often reflect radiation-induced damage. Comparing across treatment conditions, no statistically significant differences were measured in the concentration of white blood cells (WBC), red blood cells (RBC), hemoglobin (HGB), or platelets (PLT) (**Figures 1A–D**). Furthermore, there were no significant differences in the proportion of lymphocytes (%LYM) or monocytes (%MON) (**Figures 1E, F**). A statistically significant increase was observed in the proportion of neutrophils (%NEU) when comparing untreated samples to those exposed to 10 mGy of radiation (**Figure 1G**). Specifically, the mean proportion of circulating neutrophils roughly doubled, increasing from 3.71±1.73 to 7.18± 2.06 (adjusted p-value 0.041). This change was also reflected in the absolute neutrophil counts (**Supplementary Figure 1**). However, the increase in neutrophils was not significant comparing between untreated and 100 mGy, though a trend was observed (**Figure 1G**).

### Stability of splenic, thymic, and bone marrow cell populations following low-dose radiation

Next, the spleen, thymus, and bone marrow were analyzed using flow cytometry to investigate the impact of radiation exposure on various cell populations in these immune organs. Given that ionizing radiation can induce cell death, sample viability was assessed post-isolation using flow cytometry. Across all exposure conditions, no significant differences in sample viability were observed, indicating that the radiation treatment did not affect the proportion of live cells in these organs (data not shown). However, this analysis may underestimate cell death if nonviable cells were preferentially lost during tissue processing.

**Figure 2:**
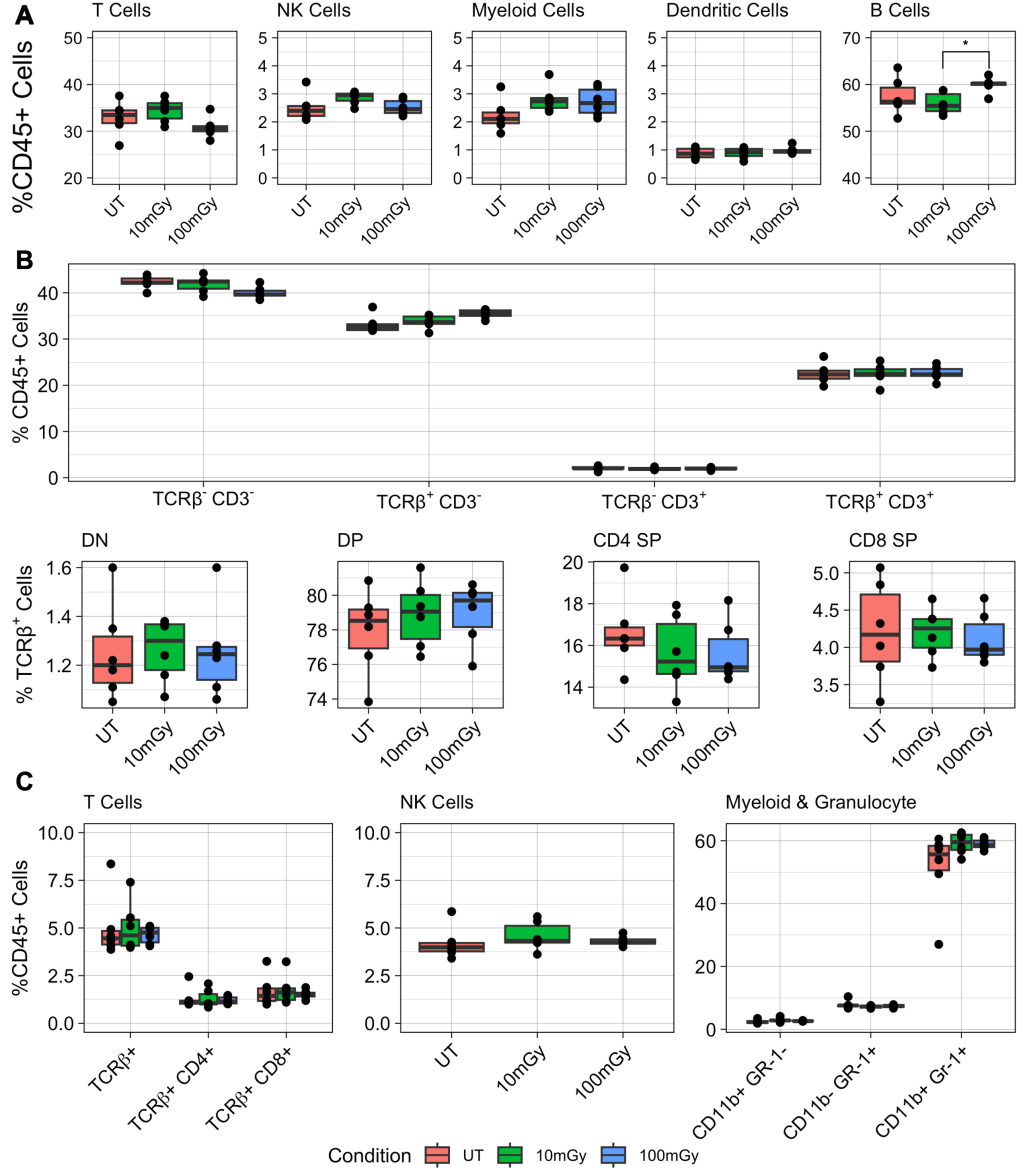
Proportion of immune cells in the spleen, thymus and bone marrow of irradiated mice. Following irradiation, immune cells were isolated from the spleen, thymus, and bone marrow, then surface-stained for flow cytometry analysis. Flow cytometry analysis of immune cell populations from mice exposed to low-dose radiation. **(A)** Percentages of immune cell subsets in the spleen: T cells (TCRβ^+^), NK cells (TCRbeta;^-^NKp46^+^), B cells (TCRβ^-^CD19^+^), Myeloid cells (CD11b^+^), Dendritic cells (CD11c^+^). **(B)** Distribution of T cell subsets in the thymus, including double-negative (DN), double-positive (DP), CD4 single-positive (CD4 SP), and CD8 single-positive (CD8 SP) populations. **(C)** Frequencies of immune cell subsets in the bone marrow, highlighting T cells (TCRβ^+^, NK cells (TCRβ^-^DX5^+^), and Myeloid and Granulocyte populations (CD11b^+^ & Gr-1^+^). Kruskal-Wallis test was used to compare means with *post-hoc* Dunn’s tests.

In the spleen, radiation exposure exerted no major effect on the proportion of immune cell subsets (**Figure 2A**). Among CD45+ cells, the proportions T cells (TCRβ+), NK cells (TCRβ- NKp46+), myeloid cells (CD11b+), and dendritic cells (CD11c+) showed no significant differences across conditions (**Figure 2A**). Although no significant changes were detected in the proportion of B cells (TCRβ- CD19+) when comparing untreated against treated samples, a minor but statistically significant difference was observed between the 10 mGy and 100 mGy conditions (adjusted p-value = 0.039) (**Figure 2A**). Overall, flow cytometry analysis of splenocytes did not show any perturbations caused by radiation exposure.

Comparing the proportions of CD3+ and TCRβ+ cells in the thymus, no significant differences were measured across all treatment conditions (**Figure 2B**). Additionally, no significant differences in the proportion of thymocytes (gated on TCRβ+) occupying each developmental stage were measured. Upon analyzing the bone marrow, no significant differences were observed in the proportion of both CD4+ and CD8+ T cells (TCRβ+) (**Figure 2C**). Similarly, the frequency of NK cells (TCRβ- DX5+) remained consistent, with no measurable variation between the experimental groups. Finally, no significant differences were found in the proportion of myeloid and granulocytes expressing CD11b or Gr-1. In summary, flow cytometry results indicate that sustained radiation exposure did not significantly alter the proportions of various immune cell subsets in the spleen, thymus, or bone marrow, suggesting a lack of significant immunological perturbations in these organs.

**Figure 3:**
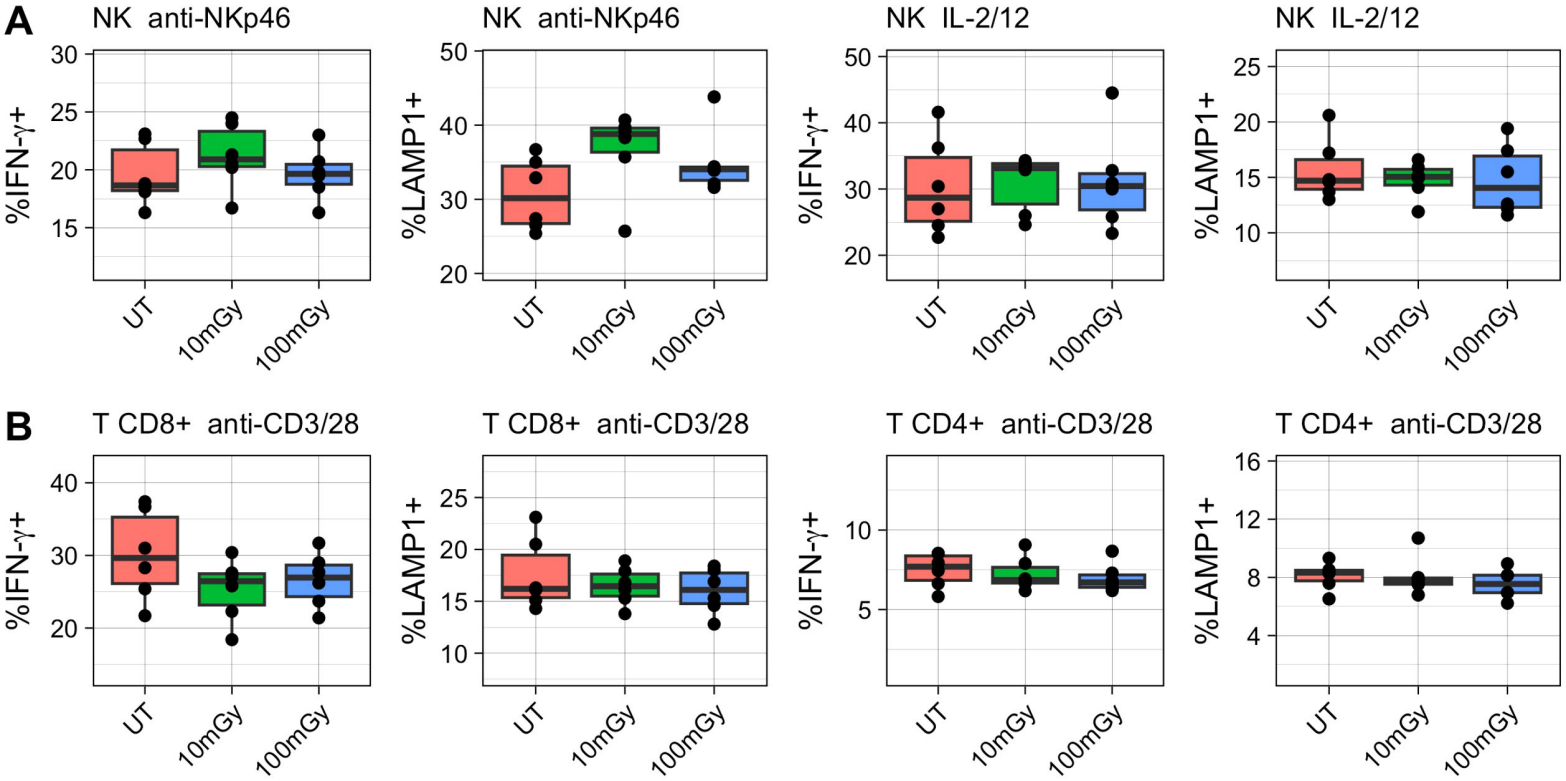
Ex vivo stimulation of irradiated immune cells. Following irradiation, immune cells were isolated from the spleen for analysis. **(A)** Natural Killer (NK) cells were stimulated with either plate coated anti-NKp46 or a combination of IL-2 and IL-12. **(B)** CD8+ and CD4+ T cells were stimulated with CD3/CD28. LAMP-1+ and IFN-γ positive cells were measured using flow-cytometry. Kruskal-Wallis test was used to compare means with post-hoc Dunn’s tests.

To determine whether LDIR exposure modulated immune cell functionality, splenic NK and T cells were stimulated ex vivo and assessed for activity based on the upregulation of interferon-gamma (IFN-γ) and CD107a (LAMP-1). When NK cells were stimulated with either anti-NKp46 or a combination of IL-2 and IL-12, no statistically significant differences were measured in the proportions of IFN-γ or LAMP-1 expressing cells across treatment conditions (**Figure 3A**). Similarly, CD8+ and CD4+ T cells displayed no differences in their response to anti-CD3 and anti-CD28 stimulation in terms of INF-*y* production (**Figure 3B**). These findings suggest that LDIR does not impact cytokine production or degranulation of NK or T cells under the experimental conditions tested.

### Minimal transcriptomic response of splenocytes to sustained low-dose radiation exposure

**Figure 4:**
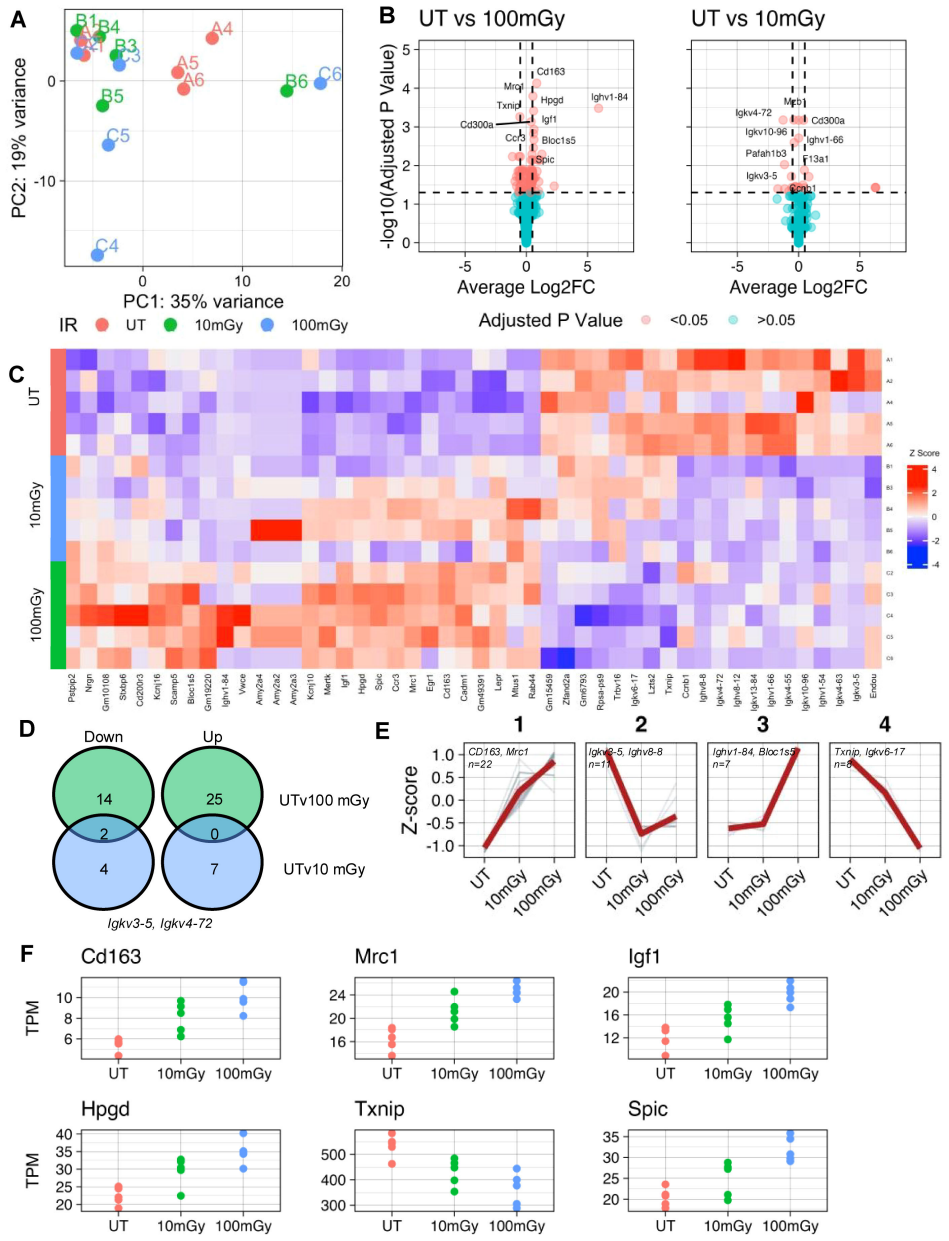
Differential expression analysis of splenocytes isolated from irradiated mice. Pairwise differential expression analysis was conducted comparing untreated (UT) samples with those exposed to either 10 mGy or 100 mGy doses. **(A)** Principal component analysis (PCA) was employed to visualize the variation in transcriptomes across samples. Treatment replicates were randomly distributed with no discernible organization by radiation treatment. **(B)** Volcano plots showing significantly differentially expressed genes (DEGs), highlighted in red (adjusted p value < 0.05). **(C)** Hierarchically clustered heatmap of significant DEGs across samples, with expression values displayed as gene wise z scores. **(D)** Venn diagram comparing significant DEGs identified in the UT vs 10 mGy and UT vs 100 mGy contrasts. **(E)** Hierarchical clustering was performed on z-score–normalized mean expression across exposure. Gene names are labeled for two representative genes with the total number of differentially expressed genes are indicated for each cluster **(F)** Expression (TPM: transcripts per million) of selected DEGs. Unless otherwise indicated, significance thresholds were defined as adjusted p value < 0.05 and |log2 fold change| > 0.5.

Next, we employed bulk RNA-sequencing to explore transcriptome-wide changes, aiming to detect subtle shifts in gene expression that might not be reflected in the limited dimensionality of flow cytometry. Principal component analysis (PCA) was employed to visualize the variation between sample transcriptomes (n=5 mice per condition) (**Figure 4A**). Treatment replicates displayed a random distribution across the two axes, with no discernible organization by radiation treatment. This observation suggests that the dominant source of transcriptomic variation in the data was not attributable to radiation exposure. Consistent with the low-dose nature of the exposure, these results suggest an absence of large-scale transcriptomic perturbation following radiation treatment.

Next, to investigate genes involved in radiation response, we performed paired differential expression analysis comparing untreated samples with those from mice exposed to either 10 mGy or 100 mGy doses (**Figure 4B**). These analyses identified a limited number of differentially expressed genes (DEGs) (adjusted p-value < 0.05). Specifically, we found 24 DEGs when comparing UT with 10 mGy and 92 DEGs when comparing UT with 100 mGy. It is noteworthy that most DEGs exhibited small changes in magnitude. Only 11 and 39 DEGs had an absolute log_2_-fold change above a cut-off of 0.5 when comparing UT to 10 mGy and 100 mGy, respectively (**Figure 4C**). The median log_2_ fold changes were 0.46 and 0.47, comparing UT to 10 mGy and 100 mGy, respectively. Among these DEGs, only two were consistently differentially expressed in both comparisons (**Figure 4D**). Hierarchical clustering of the 48 unique DEGs (adjusted p-value < 0.05 and |log_2_ fold change| > 0.5 in at least one comparison) identified three distinct transcriptomic response trajectories across doses (**Figures 4E, F**). Together, these DEGs represent candidate genes potentially modulated in immune cell responses to LDIR exposure.

To dissect functional roles of these subtly modulated genes, all significant DEGs (adjusted p-value < 0.05) were inputted into a Gene Set Enrichment analysis (GSEA). Specifically, GSEA was performed using a list of all genes ranked by their fold change when comparing untreated and treated samples. When comparing UT vs 100 mGy using the MsigDB hallmark collection and GO Biological Processes collection, 5 gene sets were significantly enriched (adjusted p-value < 0.05) (**Supplementary Figure 2A**). Across all differentially expressed genes involved in these pathways, none exhibited a log_2_ fold change exceeding 0.25. There were no enriched pathways when performing GSEA on genes ranked by differential expression comparing UT vs 10 mGy conditions.

Finally, a targeted examination of genes implicated in responses to ionizing radiation, particularly those related to DNA damage response, cell cycle regulation, and antioxidant defenses, was conducted (**Supplementary Figure 2B**). This focused investigation revealed that, with the exception of *Egr1* and *Ccnb1*, none of these historically pertinent genes were significantly differentially expressed when comparing untreated samples with either radiation treatment group. *Egr1*, a transcription factor, was significantly upregulated when comparing UT samples to 100 mGy (**Supplementary Figure 2C**). The *Ccnb1 gene*, encoding cyclin B1, was significantly downmodulated only when comparing UT with 10 mGy samples. In general, this focused analysis indicates canonical radiation responses, such as DDR or antioxidant pathways, are not substantially activated following cumulative exposure to either 100 mGy or 10 mGy *y*-radiation.

Together, these bulk RNA-sequencing results highlight the minimal impact of sustained low-dose radiation on splenocyte gene expression, an important finding that points to a limited capacity of sustained low-dose exposure to reprogram immune transcriptional states.

## Discussion

Observations from epidemiological and experimental studies indicate that exposure to LDIR can be immunomodulatory, influencing the proportion, phenotype and function of immune cells across various organs.

While high-dose radiation induces dramatic cell death seen in acute radiation syndrome (ARS), the mechanisms underlying responses in the low-dose range (<100 mGy) remain incompletely understood, particularly under sustained low-dose-rate conditions. Here, we conducted a controlled study to elucidate the effects of sustained low-dose-rate total-body irradiation (TBI) on the immune system of healthy female C57BL/6 mice, delivering cumulative absorbed doses of 10 mGy and 100 mGy over 7 days. Given the limited sample size feasible for this work, we restricted our study to female mice in order to maximize power for detecting dose-dependent effects while minimizing sex-related variability.

Our results demonstrate that sustained LDIR at these levels induces at most, only subtle changes to immune homeostasis. Analyzing the mice immediately after the 7-day sustained irradiation period, we found that hematological parameters remained comparable to those of unexposed controls, with no significant alterations in radiation-sensitive populations such as lymphocytes. The sole exception was a statistically significant doubling of circulating neutrophil proportion (and absolute count) at 10 mGy, which trended but did not reach significance at 100 mGy. This isolated finding may reflect transient radiation-induced neutrophil activation, mobilization, or altered margination/trafficking, rather than enhanced granulopoiesis, given the absence of bone marrow neutrophil expansion. However, cautious interpretation is warranted, as it could represent variability or an artifact in this cohort. Flow cytometry revealed no major perturbations in immune cell subset proportions in the spleen, thymus, or bone marrow. Ex vivo functional assays further showed preserved NK and T cell responses (IFN-γ production, degranulation), indicating no impairment or enhancement in cytokine production or effector activity under these conditions.

Bulk RNA-sequencing reinforced that the immune system was largely unperturbed by either radiation exposure. Principal component analysis showed no treatment-driven clustering, and differential expression analysis identified only modest numbers of DEGs, mostly with small fold changes. Targeted inspection of radiation-response genes (DNA damage response, cell cycle, antioxidants) showed no broad activation, except for Egr1 upregulation (at 100 mGy) and Ccnb1 downregulation (at 10 mGy). These findings suggest that the sustained LDIR tested does not strongly trigger canonical stress pathways. The minor transcriptomic shifts observed here, while statistically significant in some cases, raise questions of biological relevance due to small magnitudes, limited pathway coherence, and absence of canonical signatures. Nonetheless, subtle changes could represent sensitive adaptive responses to mild stress, and candidates like Egr1 warrant validation as potential low-dose markers in future work.

Although our study did not define a formal dose-response or dose-rate-response curve, the absence of discernible perturbations across hematological, cellular, functional, and transcriptomic readouts suggests that these exposure conditions may fall below the threshold of biomolecular insult required to elicit detectable systemic immune responses. This does not imply a threshold for DNA damage response, which is generally considered to follow a more linear relationship at low doses (32, 33). Instead, our results highlight a potential dissociation between molecular-level events and broader physiological outcomes, where efficient repair, adaptation, or homeostatic mechanisms may prevent subtle insults from translating into observable immunological changes. This complexity illustrates the challenges of extrapolating from molecular damage to whole-system effects under low-dose-rate conditions. Nevertheless, our data cannot exclude low-dose hypersensitivity or other non-linear responses at specific doses below or between 10 and 100 mGy.

Several studies using acute gamma-ray exposure have reported immune alterations following low-dose ionizing radiation (LDIR). For example, Maccullum et al. revealed that even a low-dose of 0.01 Gy prompted detectable p53 induction in the spleen and liver, with levels increasing dose-dependently (18). Similarly, Shimura et al. found upregulation of Nrf2 in PBMCs of mice exposed to acute TBI as low as 0.1 Gy (19). These results suggest an extremely sensitive mechanism in mammalian cells for detecting and responding to ionizing radiation, challenging the necessity for a threshold to trigger such responses. TBI exposure, even at 0.1 Gy could reduce total white blood cells and lymphocytes just one day after exposure (19). In the study by Bogdandi et al., mice exposed to acute TBI at 0.1 Gy and below showed most splenocyte populations experienced statistically significant reductions in cell numbers at least at one time point, with a general downward trend even when changes weren’t significant (21). Furthermore, 0.1 Gy exposure caused total splenocytes to be reduced by around 35% while 0.01 Gy led to more minor changes, with total splenocytes being reduced by around 10%. While these reports reveal immune fluctuations in the low-dose range, they all employed acute, high-dose-rate delivery. In contrast, our model delivered cumulative doses of 10 and 100 mGy at a low dose rate over 7 days. This temporal spreading of exposure likely limits the number of cells simultaneously experiencing radiation insult at the time of analysis, thereby reducing the likelihood of detecting robust signatures of DNA damage response, apoptosis, or oxidative stress compared with acute high-dose-rate models. Furthermore, any cumulative damage of radiation on the cellular environment may have been beneath a threshold necessary to activate significant immune cell responses across these organs.

Comparable studies of sustained or chronic LDIR effects on the immune system remain limited. In prior work from our group, chronic exposure to tritiated drinking water was used to investigate immune function in a transgenic breast cancer mouse model, differing from the present study’s focus on gamma radiation in healthy mice (34). More recently, we exposed female MMTV-neu transgenic mice (FVB/N background) to sustained low-dose-rate ^60^ Co gamma radiation over 56 days, achieving cumulative absorbed doses of 10 and 100 mGy (35). That study revealed modest hematological changes, altered splenic macrophage proportions, and transient increases in NK cell frequency, NKG2D expression, and ex vivo IFN-γ production, without significant effects on mammary tumorigenesis. The contrasting outcomes, minimal perturbation in the current healthy C57BL/6 model versus selective, transient innate immune activation in the transgenic model, likely reflect the much longer exposure duration (56 vs. 7 days), strain-specific differences in immune baselines and radiation sensitivity (C57BL/6 vs. FVB/N), and the influence of ongoing mammary tumorigenesis, which may modulate radiation-induced immune responses.

Other investigations of protracted gamma exposure similarly report limited immune disruption. Shin et al. investigated the effects of protracted low dose gamma radiation exposure in mice, applying a low-dose-rate of 0.7 mGy/h for 11.9 days to reach a cumulative dose of 0.2 Gy (25). They also exposed another group of mice to 3.95 mGy/h for 21 days, totaling 2 Gy. The authors reported no significant changes in hematological metrics at either dose rate. Additionally, they found no differences in splenocyte population proportions, even with a cumulative 2 Gy dose, contrasting with findings from Bogdandi et al., where 2 Gy delivered acutely caused substantial shifts in splenocyte populations (21). They did, however, reveal minor fluctuations (under 0.5-fold) in various cytokines. In a unique study by Courtade et al., mice received cumulative 20 cGy whole-body gamma irradiation over 24 months (36). On a per-day basis, this rate is approximately 0.0274 mGy/day. The results showed that this chronic low-dose exposure did not significantly alter the total counts of splenocytes or thymocytes, although a small, temporary increase in splenocyte and thymocyte counts was observed at 12 and 18 months, respectively. T-cell populations in both the spleen and thymus showed no notable changes, indicating that cellular immunity was not strongly affected by this dose rate. Immunoglobulin levels, however, showed a dose-dependent decline over time, although B-cell numbers in the spleen remained stable. In general, evidence suggests that low-dose-rate radiation is less perturbing to the immune system compared to the same dose given at a high-dose rate. Low-dose-rate radiation given to a low cumulative dose is very minimally affecting, rarely influencing immune cell proportions or phenotypes. Comparatively, low-dose-rate radiation given to higher cumulative doses may be sufficient to trigger systemic immune system responses, with variation depending on the radiation dose, quality and rate as well as the mouse strain, age, and sex (37).

Despite the comprehensive nature of our analysis, our study has important limitations. The sample size was small, which may limit the statistical power to detect the subtle changes expected from LDIR exposure. Furthermore, incorporating measurements of DNA damage following radiation exposure, such as γ-H2AX staining, would have provided more direct evidence of radiation-induced DNA damage, thereby contextualizing our findings. Another limitation is that only female mice were studied. This choice was deliberate, as our sample size was insufficient to robustly evaluate sex differences, and restricting to females allowed for more straightforward interpretation of dose-dependent effects. However, this necessarily limits generalizability to males. Additionally, our study examined the effects immediately after exposure, yet the potential impacts at later stages remain uncertain. Lastly, since factors such as model organisms, strains, age, sex, and varying radiation quality and quantity can significantly impact biological outcomes, the findings from this study may have limited generalizability. The convergence of results from hematology, flow cytometry, and transcriptomics provides a consistent and comprehensive description of the lack of measurable radiation response, thereby enhancing the credibility of our conclusions.

Importantly, major epidemiological studies have shown excess relative risk scores for cancer and non-cancer diseases among occupationally exposed nuclear energy workers (NEW) (12, 14–16, 38–40). According to the Canadian Nuclear Safety Commission, a typical annual dose received by a NEW in a uranium mine or nuclear power plant in Canada is around 1 mSv (41). In a publication from the million-person study analyzing US NEWs, the mean cumulative career dose was 52.6 mSv, with a maximum dose of 1.32 Sv and only around 15% of workers with career exposures over 100 mSv (40). In contrast, the cumulative doses and dose rates used in our study are significantly higher than those typically encountered by NEWs. Nevertheless, our results demonstrate that even at the upper threshold of what is considered a low-dose (100 mGy), we found little to no evidence of immune perturbation in female mice. Direct extrapolation to human health risks remains limited due to species differences, exposure duration, genetic background, sex, and endpoint timing; further studies bridging these gaps are needed to inform radiation protection frameworks.

Future studies should build on these findings by establishing comprehensive dose-response curves for LDIR effects on immune readouts, extending across the low-dose range (e.g., <100 mGy) into higher-dose regimes to delineate potential thresholds, adaptive responses, or non-linear transitions where perturbations become more pronounced. Extending exposure durations beyond the 7-day period used here, such as weeks to months of sustained low-dose-rate irradiation, will be essential to better model chronic occupational or environmental scenarios and to capture potential cumulative or time-dependent immunological adaptations. Additionally, experimental designs that more closely simulate human exposure conditions, including varied dose rates, fractionated delivery, and diverse genetic backgrounds, age groups, or sexes, would enhance translational relevance. Additionally, identifying radiation biomarkers would be extremely valuable for future LDIR epidemiology. Such biomarkers could help researchers distinguish between radiation-induced health effects and background variations, thereby contributing to reducing the current uncertainties in epidemiological surveys at low dose exposures and contributing to the generation of radiation dose response models.

## Conclusion

This study investigated the effects of sustained LDIR on immune cell composition and gene expression in a murine model. Female C57BL/6 mice exposed to continuous low-dose-rate ^60^Co gamma radiation to cumulative doses of 10 mGy and 100 mGy displayed limited alterations in blood composition or immune cell distributions across the spleen, thymus, and bone marrow. Transcriptomic analysis revealed only minimal gene expression differences, with no detection of canonical radiation response pathways, such as DNA damage repair or oxidative stress. These findings suggest that, under the conditions studied, the female murine immune system remains largely unperturbed by sustained low-dose radiation, even at cumulative doses at the upper threshold for low-dose exposure as defined by the US National Academies of Science.

## Data Availability

The datasets presented in this study can be found in online repositories. The names of the repository/repositories and accession number(s) can be found below: https://www.ncbi.nlm.nih.gov/geo/, GSE296450.

## Supplementary material

Supplementary Figure 1 Supplemental Hematology Results. Complete blood count (CBC) parameters following low-dose irradiation. Mice were either untreated (UT) or exposed to 10 or 100 mGy total body irradiation. Boxplots show **(A)** lymphocytes (LYM), **(B)** monocytes (MON), **(C)** neutrophils (NEU), **(D)** hematocrit (HCT), **(E)** mean corpuscular volume (MCV), **(F)** mean corpuscular hemoglobin (MCH), **(G)** mean corpuscular hemoglobin concentration (MCHC), **(H)** red cell distribution width–standard deviation (RDW-SD), **(I)** red cell distribution width–coefficient of variation (RDW-CV), **(J)** platelet count (PLT), **(K)** plateletcrit (PCT), **(L)** mean platelet volume (MPV), **(M)** platelet distribution width–standard deviation (PDW-SD), and **(N)** platelet distribution width–coefficient of variation (PDW-CV). Kruskal-Wallis tests were used to compare the means of each parameter with a *post-hoc* Dunn’s test being applied to test pairwise comparisons. Asterix represents an adjusted p-value < 0.05.

Supplementary Figure 2 Inspection of Radiation Response in Bulk RNA-Seq. **(A)** GSEA was performed by ranking all DEGs by fold change from the untreated (UT) vs. 100 mGy comparison and comparing them to hallmark pathways from the MSigDB mouse-specific reference. Pathways with significant enrichment (adjusted p-value < 0.05) are displayed, with bar color intensity representing the adjusted p-value. No significant pathways were found for the 10 mGy dose. **(B)** List of genes implicated in the response to ionizing radiation was obtained from the Gene Ontology database. A clustered heatmap plotting the z-score of the radiation pathway genes demonstrates minimal discernible organization of their expression across treatment replicates. **(C)** Expression of Egr1 and Ccnb1 across conditions in transcripts per million (TPM). Significant differential expression of Egr1 was found when comparing UT vs. 100 mGy. Ccnb1 was significantly modulated when comparing UT vs. 10 mGy. Asterisk denotes adjusted p-value < 0.05.
