# Correction: Limited immune perturbations in mice exposed to sustained low-dose ionizing radiation

**DOI:** 10.3389/fimmu.2026.1880777

**Published:** 2026-06-03

**Authors:** Bryan Marr, Holly Laakso, Melinda Blimkie, Andrew Cao, Seung-Hwan Lee, Abrar Ul Haq Khan

**Affiliations:** 1 Department of Biochemistry, Microbiology, and Immunology, Faculty of Medicine, University of Ottawa, Ottawa, ON, Canada; 2 Radiobiology and Health Branch, Canadian Nuclear Laboratories Ltd., Chalk River, ON, Canada; 3 Ottawa Institute of Systems Biology, Faculty of Medicine, University of Ottawa, Ottawa, ON, Canada; 4 Centre for Infection, Immunity, and Inflammation, Faculty of Medicine, University of Ottawa, Ottawa, ON, Canada

**Keywords:** ionizing radiation, low-dose radiation, low-dose rate, radiation immunology, RNA-seq

There was a mistake in **Figure 4** as published. In **Figure 4B** both volcano plots of 10 mGy and 100 mGy are same and accidentally duplicated. The corrected **Figure 4** appears below. The correct image presented here was among the files assessed in peer review, but was accidentally replaced by the duplicate image in a subsequent revision.

**Figure 4:**
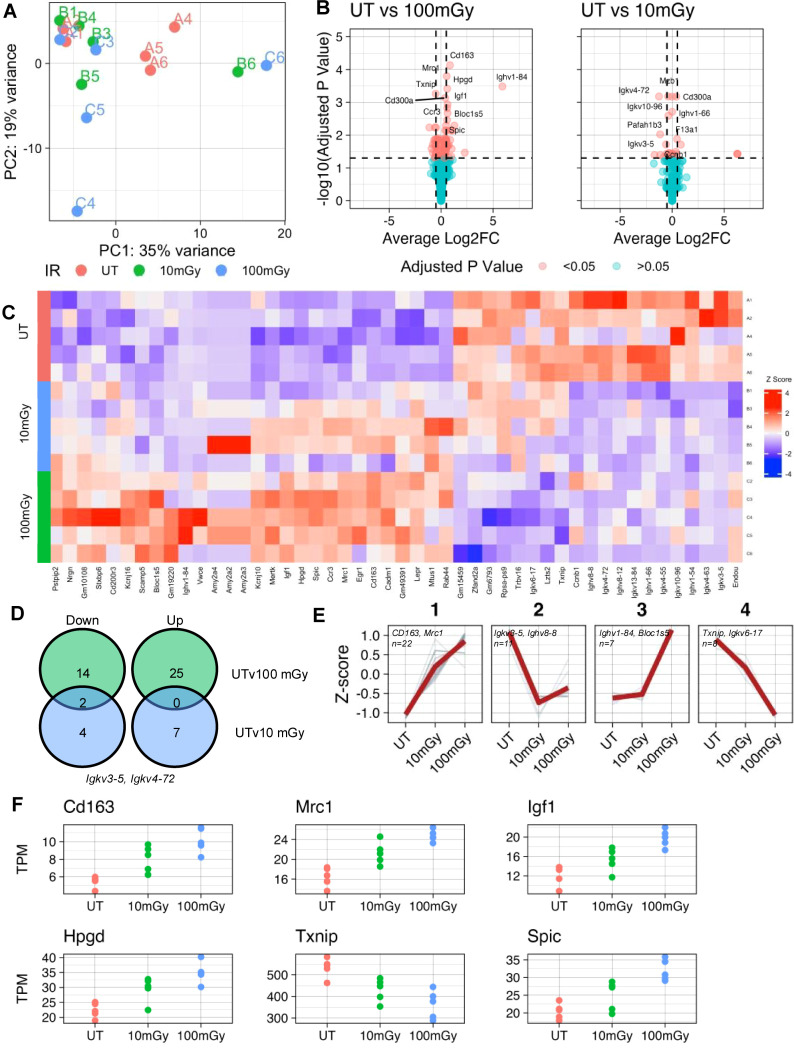
Differential expression analysis of splenocytes isolated from irradiated mice. Pairwise differential expression analysis was conducted comparing untreated (UT) samples with those exposed to either 10 mGy or 100 mGy doses. **(A)** Principal component analysis (PCA) was employed to visualize the variation in transcriptomes across samples. Treatment replicates were randomly distributed with no discernible organization by radiation treatment. **(B)** Volcano plots showing significantly differentially expressed genes (DEGs), highlighted in red (adjusted p value < 0.05). **(C)** Hierarchically clustered heatmap of significant DEGs across samples, with expression values displayed as gene wise z scores. **(D)** Venn diagram comparing significant DEGs identified in the UT vs 10 mGy and UT vs 100 mGy contrasts. **(E)** Hierarchical clustering was performed on z-score–normalized mean expression across exposure. Gene names are labeled for two representative genes with the total number of differentially expressed genes are indicated for each cluster **(F)** Expression (TPM: transcripts per million) of selected DEGs. Unless otherwise indicated, significance thresholds were defined as adjusted p value < 0.05 and |log2 fold change| > 0.5.

The original version of this article has been updated.

