# Supplementary figures and images for: Limited immune perturbations in mice exposed to sustained low-dose ionizing radiation
Source: Front Immunol. 2026 Apr 20;17:1642012. doi: 10.3389/fimmu.2026.1642012 (PMC13136176; doi:10.3389/fimmu.2026.1642012)

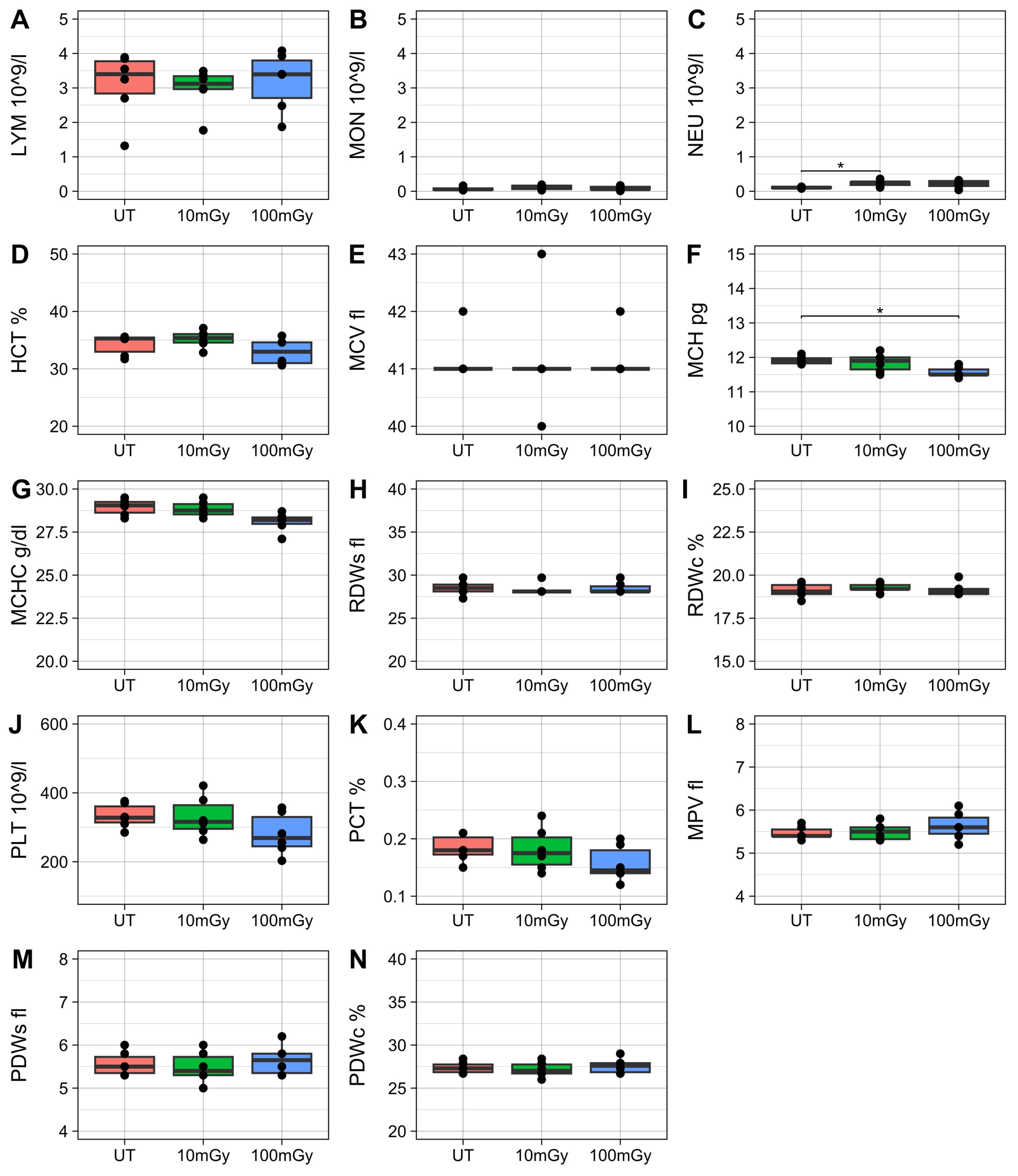

Supplement: Supplementary Figure 1 — Supplemental Hematology Results. Complete blood count (CBC) parameters following low-dose irradiation. Mice were either untreated (UT) or exposed to 10 or 100 mGy total body irradiation. Boxplots show (A) lymphocytes (LYM), (B) monocytes (MON), (C) neutrophils (NEU), (D) hematocrit (HCT), (E) mean corpuscular volume (MCV), (F) mean corpuscular hemoglobin (MCH), (G) mean corpuscular hemoglobin concentration (MCHC), (H) red cell distribution width–standard deviation (RDW-SD), (I) red cell distribution width–coefficient of variation (RDW-CV), (J) platelet count (PLT), (K) plateletcrit (PCT), (L) mean platelet volume (MPV), (M) platelet distribution width–standard deviation (PDW-SD), and (N) platelet distribution width–coefficient of variation (PDW-CV). Kruskal-Wallis tests were used to compare the means of each parameter with a post-hoc Dunn’s test being applied to test pairwise comparisons. Asterix represents an adjusted p-value < 0.05. [file Image1.jpeg]

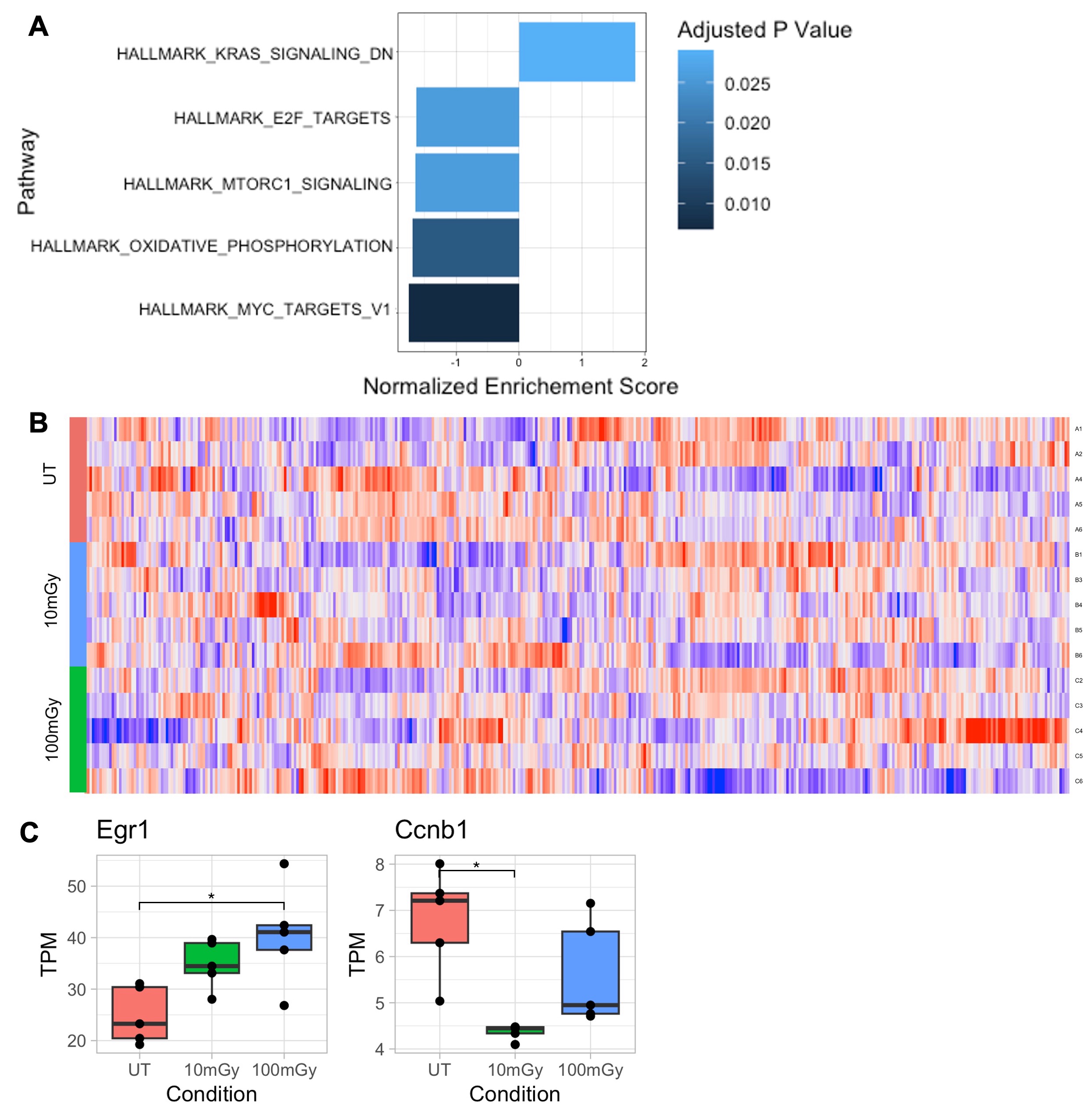

Supplement: Supplementary Figure 2 — Inspection of Radiation Response in Bulk RNA-Seq. (A) GSEA was performed by ranking all DEGs by fold change from the untreated (UT) vs. 100 mGy comparison and comparing them to hallmark pathways from the MSigDB mouse-specific reference. Pathways with significant enrichment (adjusted p-value < 0.05) are displayed, with bar color intensity representing the adjusted p-value. No significant pathways were found for the 10 mGy dose. (B) List of genes implicated in the response to ionizing radiation was obtained from the Gene Ontology database. A clustered heatmap plotting the z-score of the radiation pathway genes demonstrates minimal discernible organization of their expression across treatment replicates. (C) Expression of Egr1 and Ccnb1 across conditions in transcripts per million (TPM). Significant differential expression of Egr1 was found when comparing UT vs. 100 mGy. Ccnb1 was significantly modulated when comparing UT vs. 10 mGy. Asterisk denotes adjusted p-value < 0.05. [file Image2.jpeg]
